# MoGAAAP: a modular Snakemake workflow for automated genome assembly and annotation with quality assessment

**DOI:** 10.1093/nargab/lqag008

**Published:** 2026-01-22

**Authors:** Dirk-Jan M van Workum, Kuntal K Dey, Alexander Kozik, Dean O Lavelle, Dick de Ridder, M Eric Schranz, Richard W Michelmore, Sandra Smit

**Affiliations:** Bioinformatics Group, Wageningen University & Research, Droevendaalsesteeg 1, 6708 PB, Wageningen, the Netherlands; Genome Center and Department of Plant Sciences, University of California, Davis, 451 Health Sciences Drive, Davis, CA 95616, United States; Genome Center and Department of Plant Sciences, University of California, Davis, 451 Health Sciences Drive, Davis, CA 95616, United States; Genome Center and Department of Plant Sciences, University of California, Davis, 451 Health Sciences Drive, Davis, CA 95616, United States; Bioinformatics Group, Wageningen University & Research, Droevendaalsesteeg 1, 6708 PB, Wageningen, the Netherlands; Biosystematics Group, Wageningen University & Research, Droevendaalsesteeg 1, 6708 PB, Wageningen, the Netherlands; Genome Center and Department of Plant Sciences, University of California, Davis, 451 Health Sciences Drive, Davis, CA 95616, United States; Bioinformatics Group, Wageningen University & Research, Droevendaalsesteeg 1, 6708 PB, Wageningen, the Netherlands

## Abstract

With the current speed of sequencing, there is a desire for standardized and automated genome assembly and annotation to produce high-quality genomes as input for comparative (pan)genomics. Therefore, we created a convenience pipeline using existing tools that creates annotated genome assemblies from HiFi (and optionally ultra-long ONT and/or Hi-C) reads for a set of related individuals as well as a related reference genome. Our pipeline is species-agnostic and generates an extensive quality assessment report that can be used for manual filtering and refinement of the assembly and annotation. It includes statistics for individual completeness and contamination assessments as well as a concise pangenome view. The pipeline is implemented in Snakemake and available with a GPLv3 licence at GitHub under github.com/dirkjanvw/MoGAAAP, at Zenodo under doi.org/10.5281/zenodo.14833021, and can be installed through Bioconda.

## Introduction

With the increase in DNA sequencing throughput, read length, and base-calling accuracy, the bottleneck in genomics research has shifted from data generation to data analysis. Sequencing the first human genome took many years, whereas a human genome can currently be sequenced in just a few days and assembled in approximately half a day. Pacific Biosciences HiFi and Oxford Nanopore (ONT) long-read sequencing technologies now make it possible to generate whole genome assemblies for many individuals per species. Building on existing high-quality reference genomes, efficient workflows are needed to create annotated assemblies for these additional individuals of the same species.

The genome assembly process itself has improved over the years with a focus on novel algorithms for assembly and integration of multiple types of input data. For complex eukaryotic genomes, subsequent structural and functional annotation is still a laborious process; accurate, *de novo* annotation of a genome assembly (also involving repeat masking) typically takes up to a week for computation alone, with additional manual evaluation and filtering. However, to annotate successive genome assemblies for a species, faster approaches, such as the transfer of annotations from reference genomes and *ab initio* predictions based on machine learning, are available.

Here, we address the need for both (i) increasing the speed of creating annotated genome assemblies while decreasing the effort required and (ii) standardizing the output, necessary for downstream comparative (pan)genomics. To this end, we implemented MoGAAAP, an automated workflow using a modular Snakemake pipeline that fully automates the processes of assembly, provisional annotation, and quality assessment (QA) for any diploid eukaryotic organism, thereby making the process of genome assembly and annotation more accessible to a wider audience. MoGAAAP does not aim to create a perfect, publication-quality genome assembly but rather to provide a high-quality foundation for further refinement, if required. Also, due to its modular nature, MoGAAAP can be used for only assembly, annotation, or QA of already existing genomes in any combination. The pipeline creates a standardized QA report, which provides the user with suggestions for additional (manual) curation and filtering of the input reads, assembly, and annotation. Such automation of these processes will significantly increase the speed of exploring genome repertoires of diverse eukaryotic species.

## Implementation

**Figure 1.**
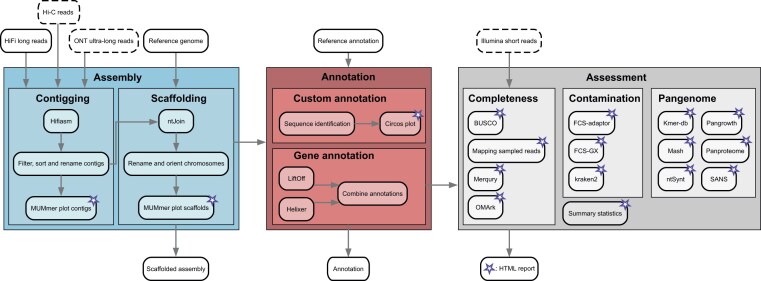
Schematic overview of the MoGAAAP pipeline. The pipeline consists of three main parts, which are subdivided into multiple modules. The main input and output files are highlighted above and below the modules, respectively. Input files in dashed boxes are optional. All rules of the pipeline that create output for the final reporting are highlighted with a star.

We created a generalized modular Snakemake ($\ge$v8.0) pipeline [1] that takes as input HiFi (and optionally ONT and/or Hi-C) reads as well as a chromosome-level, annotated reference genome, and creates an annotated genome assembly, including a QA report (Fig. 1). The quality of the resulting assembly strongly depends on coverage and quality of the input data. The overall process takes ~2 days for a human genome; however, the exact runtime depends on genome size and available computational resources. Because of the modularity of MoGAAAP, the starting point of an analysis can also be an already created pseudomolecule-level assembly (with or without annotation). This means that MoGAAAP can also be used for QA of existing genomes.

### Assembly

#### Contigging

Input HiFi reads are assembled using hifiasm v0.25.0 [2] with default parameters. If ultra-long ONT reads are provided, they are passed on to hifiasm using the ‘-‌-‌ul’ parameter (if only ONT reads are provided, they are passed on with the ‘-‌-ont’ parameter). Hi-C reads, if provided, are also passed to hifiasm. As alternatives to hifiasm, we included both Verkko v2.1 [3] and Flye v2.9.5 [4], which demonstrates the modularity of the pipeline (future assemblers can be added). Both homozygous and heterozygous diploid genomes are supported by hifiasm, resulting in a phased assembly for heterozygous genomes. Similarly, for Verkko and Flye, HapDup v0.12 [5] is used to obtain such a phased assembly. All contigs are retrieved from the resulting assembly graph and written to FASTA format. This FASTA file can be filtered for short contigs and is sorted on size. MUMmerplot v4.0.0rc1 [6] then generates a plot that is added to the report for visual inspection of the resultant assembly compared to a predefined reference genome. The pipeline does not perform filtering of the input sequencing data, so as to reflect the content of the original input library. However, if contamination is detected through the QA module, filtering of input reads may be required to improve the assembly quality. Separation of nuclear and organellar sequences occurs in the ‘Custom annotation’ module.

#### Scaffolding

Because for many species relevant to science and society at least one high-quality chromosome-level genome assembly is now available, we implemented reference-guided scaffolding and chromosomal orientation in the pipeline, useful to efficiently scaffold additional genomes for the same species. If Hi-C data are available, MoGAAAP can use YaHS [7] for initial scaffolding prior to reference-guided scaffolding. For reference-guided scaffolding, MoGAAAP uses the minimizer-based tool ntJoin v1.1.4 [8] with user-provided values for the window and *k*-mer length, or alternatively RagTag, which is an alignment-based approach to reference-guided scaffolding [9]. The resulting chromosomes are renamed and orientated to be consistent with the supplied reference genome (obtained via a quick mashmap mapping) [10]. MUMmerplot is used again to generate a plot for the report for visual inspection of the scaffolding, renaming, and orienting processes. Hi-C reads are not used for automated scaffolding, as no currently available tool guarantees correctly scaffolded chromosomes. Instead, if Hi-C reads were provided, a contact map is generated using HapHiC’s plotting function [11] to assess the reference-guided scaffolding.

### Annotation

#### Gene annotation

Accurate and comprehensive *de novo* gene annotation, as would typically be done for a first reference genome for a species, is currently an iterative and time-consuming process, which cannot be fully automated. Therefore, we implemented a workflow that creates a provisional annotation by combining Liftoff v1.6.3 [12] and Helixer v0.3.2 [13] runs on the scaffolded assembly. Since neither tool requires repeat masking, this speeds up the process of gene annotation significantly. For Liftoff, the annotation corresponding to the high-quality chromosome-level reference assembly that was used for reference-guided scaffolding is used. Liftoff tries to transfer all annotated gene features, which may include both protein-coding and non-coding genes. Liftoff is run with the ‘-copies -cds -polish’ parameters to adjust for potentially slightly shifted gene models in the newly assembled genome. Since Liftoff may yield invalid open reading frames, we use AGAT v1.4.0 [14] to remove these. This Liftoff annotation is then supplemented with Helixer-predicted protein-coding genes, again using AGAT; in case of an overlap between Liftoff and Helixer, we favour the Liftoff prediction. Although the quality of this provisional annotation is typically high, it is not evidence-based, necessitating cautious use of the predicted genes. Transcript or protein data can be aligned to estimate the reliability of gene models. Alternatively, if resources are available, an evidence-based annotation can be produced, after which MoGAAAP could be used for quality assessment.

#### Custom annotation

Genome assemblies may be generated for the investigation of specific gene families (e.g. NLRs, transcription factors) or specific sequences (e.g. telomeres, centromeres, rDNA arrays) across genomes. These user-provided queries can be both nucleotide or protein sequences and are searched using BLAST v2.15.0 [15] against the scaffolded assembly. All these queries are used to create a Circos v0.69.9 [16] configuration file, which can be polished further to address the user’s needs outside of the pipeline. Additionally, organellar sequences are identified and separated from the ‘nuclear’ assembly based on a hit of >50% of a contig’s length to organellar genomes (separation only for final reporting, not for QA).

### Assessment of assembly and annotation quality

#### Completeness

First, Merqury v1.3 [17] is employed for calculating a consensus quality value (QV) based on HiFi reads. When Illumina reads are available, these are used for an independent check of *k*-mer completeness instead. MoGAAAP also provides a mapping report of Illumina reads for this. In addition, the pipeline runs two gene completeness assessment tools: BUSCO v5.8.0 [18] and OMArk v0.3.0 [19]. BUSCO scores have traditionally been the gold standard for gene completeness; however, in the contemporary era of HiFi assemblies, incomplete assemblies (<90% complete) have become a rarity. Completeness assessed against the larger OMA database is therefore a more informative metric of annotation completeness. Together, BUSCO and OMArk give an insight into gene completeness of the assembly and annotation.

#### Contamination

In addition to OMArk, which summarizes possible contamination in the predicted proteome, contamination of the assembly itself is assessed using two separate approaches: Kraken2 v2.1.3 [20] and FCS-GX v0.5.0 (the NCBI tool for finding potential contamination) [21]. Kraken2 (ideally run against a recent ‘nt’ database) is used for creating a Krona v2.8.1 report [22] showing the likely origin of each scaffold/contig based on *k*-mer composition. Together with the FCS-GX report, this is provided to the user for deciding which scaffolds/contigs need to be removed from the assembly.

#### Pangenome

Finally, we provide insights into a pangenome of user-defined sets of individuals (genotypes, accessions, or strains) to assess its diversity. Since typically multiple related individuals may be sequenced for assembly, pangenome completeness can provide an overview of the covered diversity of the related individuals. At the genome structure level, we use ntSynt v1.0.0 [23] to calculate collinearity between the assemblies based on minimizers (which makes it independent of gene annotation). The resulting plot can be used to identify large inversions and translocations, indicating potential misassemblies or real structural variation. Next, we employ mash [10] for calculating mash distance between the assemblies. The resulting heatmaps give insight into the phylogenetic relationships between the assemblies; this can be compared to the known evolutionary origin of the individuals for identifying potential sample swaps or mislabelling. For assessing the openness of the pangenome, we use both *k*-mer and gene-based metrics. We employ pangrowth ($@$ commit 71d67bde89326644f6718c82ec2ee7b751f3080b) [24] for calculating the pangenome growth based on *k*-mers and PanTools v4.3.1 [25] for the pangenome growth based on genes.

## Validation and application

To demonstrate and validate MoGAAAP’s applicability across diverse diploid species, we describe four use cases in Supplementary data. First, we showed the usability of the pipeline for the generation of pangenome-sized data by reassembling and analysing a pangenome of 32 *Arabidopsis thaliana* accessions, for most of which only HiFi data are available [26]. Since not all plant genomes are homozygous, we also showed the effectiveness of MoGAAAP on six highly heterozygous grapevine genomes. Next, we demonstrated the applicability of the pipeline to organisms outside of the plant kingdom by assembling a trio from the human pangenome project. To demonstrate broader applicability across eukaryotes, we also successfully used MoGAAAP for the assembly, annotation, and analysis of an invertebrate and fungal genome. Through these use cases, we found that MoGAAAP was able to replicate the findings of the original analyses. Subsequently, we applied the pipeline to a newly generated dataset of *Lactuca serriola*, a wild relative of cultivated lettuce (*Lactuca sativa*), on which both HiFi and ONT sequencing have been performed. This resulted in the first chromosome-level genome of *L. serriola*, which we made publicly available.

We found the QA to be a highly important part of the pipeline (see Supplementary data). Most assemblies were scaffolded into correct chromosomes and showed little to no contamination. Interestingly, all human genomes were processed correctly, likely because most of the tools employed were developed for the field of human genomics. For the assemblies that showed unexpected outlier statistics in the QA report, we could easily use the report to identify the cause, confirming issues such as contamination and mis-scaffolding based on the output of multiple tools. For example, we found that contamination of foreign DNA was a major factor that influenced the accuracy of the genome assembly and resulting gene annotation. Ideally, all contamination is removed from the input data before assembling the reads. Alternatively, contaminations may be removed from the genome post-assembly in case they were not integrated in any sequences. This highlights the iterative nature of the genome assembly process, which we help by providing QA that is as extensive as possible. Therefore, although the pipeline is built to enable an end-to-end automated workflow, some manual curation and filtering remains of vital importance to create a publication-quality genome assembly.

## Strengths and limitations

MoGAAAP is designed to automate and standardize the generation of genomes after the first high-quality genome for a species. It provides a workflow to go from raw input data to a quality assessment report of an assembled and annotated genome, which may be adapted by users to their own needs. Because of the modular structure of the pipeline, it is relatively straightforward to turn a module on or off, or to replace one tool with another. For example, the pipeline employs ntJoin for scaffolding the contigs according to a reference genome. However, for some species there might not be a high-quality chromosome-level reference genome available. In these cases, other methods for scaffolding need to be used instead, e.g. based on Hi-C data. The annotation module, where we lift over the genes from a supplied reference genome and add all non-overlapping genes as identified by Helixer, provides a similar case: if the species is being assembled, the annotation of the supplied reference genome is of low quality, the resulting annotation will be of low quality too. In this case, it is advisable to run an evidence-based annotation pipeline such as Maker [27], BRAKER3 [28], or EGAPx [29] instead.

The quality of the resulting assembly is dependent on the quality and quantity of the reads supplied. Ideally, the number of contigs should be in the hundreds rather than the thousands. If there are too many small contigs, reference-guided scaffolding using ntJoin will mask structural differences, falsely suggesting full collinearity with the reference genome. Therefore, only structural variation within a single contig compared to the reference genome can be detected. Comparisons of the MUMmerplots before and after reference-guided scaffolding indicate whether this is a potential constraint.

Finally, our pipeline focuses on diploid organisms; however, many organisms have a more complex genetic make-up. Since allopolyploid genomes can be treated as heterozygous diploid genomes, each haplotype is expected to assemble separately. On the other hand, organisms with autopolyploid or aneuploid genomes are currently very challenging to assemble. Since currently no satisfactory assembly tool exists for such genomes, MoGAAAP cannot produce high-quality genomes for them. In the future, it will be relatively easy to include such tools, once available, due to the modular set-up of MoGAAAP.

## Conclusion

In conclusion, we developed a general-purpose, scalable, and modular pipeline for the assembly of HiFi data, subsequent annotation, and quality assessment. In the use cases provided, we highlight the importance of QA and subsequent custom curation of the input and/or output, which remains essential to obtain publication-quality genomes. Also, we demonstrated MoGAAAP’s use from single genome assemblies to entire pangenome datasets and from small to large genome sizes. Because of MoGAAAP’s modular setup, new modules for polishing, (satellite) repeat identification, and organellar genome assembly can be added to the Snakemake pipeline in the future. Moreover, this is deemed essential to keep the pipeline state-of-the-art.

Pipelines such as MoGAAAP will be increasingly important now that the field of genomics is moving towards pangenomic analyses, which critically depend on the availability of large numbers of related high-quality genomes. A standardized pipeline that can take sequencing data to useful (visual) output in a fast, accurate, and comprehensive manner ensures data consistency and quality for downstream applications.

## Supplementary Material

lqag008_Supplemental_File

## Data Availability

The novel *L. serriola* genome, which is described as a use case in the Supplementary data, has been published at NCBI GenBank (GCA_051521515.1). Underlying HiFi and ONT sequencing data has been published under BioProject PRJNA412928. HTML reports created by MoGAAAP for the use cases described in the Supplementary data are available through data. 4TU (DOI: 10.4121/4b39da65-2eef-4e05-8583-25f8850cf932).
