# Supplementary material for: MoGAAAP: a modular Snakemake workflow for automated genome assembly and annotation with quality assessment
Source: NAR Genom Bioinform. 2026 Jan 22;8(1):lqag008. doi: 10.1093/nargab/lqag008 (PMC12824462; doi:10.1093/nargab/lqag008)
Supplement: lqag008_Supplemental_File [file lqag008_supplemental_file.pdf]

Supplementary Information

Belongs to: "MoGAAAP: A modular Snakemake workflow for automated genome assembly and annotation with quality assessment (van Workum et al., 2025)"

Contents

Motivation **1**

Validation **1**

Arabidopsis thaliana 1

Grapevine 4

Human trio 5

Silkmoth 6

Yeast 6

Application **8**

Lactuca serriola 9

Motivation

Across plant, animal and human genomics, population studies increasingly focus on obtaining high-quality genome assemblies for pangenomic studies of diversity panels. Typically, these species already have one or more reference genomes available, simplifying the generation of successive genomes for the same species based on long-read sequencing techniques such as PacBio HiFi sequencing. MoGAAAP was designed to precisely address this challenge: automate the generation of an annotated chromosome-level genome assembly with an extensive QA report for species with already one high-quality genome available.

The development of MoGAAAP was sparked initially by a sequencing project in lettuce, aiming to deliver high-quality assemblies of many genotypes, complementing a handful of available high-quality references. As similar efforts are made for many important plant species, animal species and human populations, there is great potential for a standardised workflow. While we designed the basis of MoGAAAP to assemble and annotate lettuce genomes in high throughput, we realised that the general design makes it applicable to all such eukaryotic species.

Here, we present six different use cases to demonstrate the functionality of the MoGAAAP pipeline (github.com/dirkjanvw/MoGAAAP). First, we validate the working of MoGAAAP on five use cases (two plant datasets, one human trio, one invertebrate genome and one fungal genome), after which we apply it to one of our lettuce genomes: *Lactuca serriola*. For reproducibility, we provide the input and output (HTML report) for each use case in a data repository at data.4TU (DOI: 10.4121/4b39da65-2eef-4e05-8583-25f8850cf932).

Validation

Arabidopsis thaliana

To showcase the ease of using MoGAAAP for pangenome studies, we reproduced a pangenome set of assemblies using our pipeline. Pipelines for the automated assembly, annotation and assessment are especially useful for large-scale sequencing projects to reduce the number of technical artefacts between the resulting assemblies. Here, we re-analysed the 32 *Arabidopsis thaliana* pangenome from Kang et al. (2023).

Supplementary Table 1: Key statistics of the genomes included in the *A. thaliana* pangenome from MoGAAAP. Please note that MoGAAAP did not assembly or annotate the "ARAPORT11" genome but that MoGAAAP was only used for QA of this genome.

Name	Total length	#sequences	N50	Total QV	#genes
01_col	163998262	491	26111065	55.0955 (HiFi)	41464
02_tibet	178710383	841	27183653	25.6917 (Illumina)	47579
03_yilong	199188517	925	21988047	21.7442 (Illumina)	51253
04_bor_1	142855370	215	27158302	58.2886 (HiFi)	39482
05_cdm_0	129761192	58	22284775	70.6216 (HiFi)	33381
08_kondara	135827444	89	31843699	64.5636 (HiFi)	33753
12_li_of_095	145721566	311	25528551	56.4816 (HiFi)	40874
13_got_22	148858971	310	25034424	56.9546 (HiFi)	40689
14_st_0	139874502	180	24648369	58.8654 (HiFi)	37643
15_kelsterbach_2	149571253	421	27330886	56.3584 (HiFi)	42644
19_kz_9	133427053	66	25771137	62.8139 (HiFi)	34485
20_ll_0	141988299	110	27362594	63.0672 (HiFi)	36768
21_ms_0	144019097	307	24999985	57.9345 (HiFi)	40925
23_sij_1	142192014	240	24171901	57.5884 (HiFi)	39149
24_hs_0	135757155	219	25370822	57.5047 (HiFi)	35734
25_per_1	144712708	257	25483084	58.1671 (HiFi)	39532
26_nz_1	139076256	51	27091447	66.3403 (HiFi)	34765
27_belmonte_4_94	132508182	49	26605651	70.2931 (HiFi)	34415
29_sij_2	135814710	51	25116981	66.6536 (HiFi)	34401
30_tu_sb30_3	166863648	782	26088619	53.7577 (HiFi)	47406
31_mammo_1	137245559	44	26735169	65.9772 (HiFi)	34578
33_sha	136711704	48	24742906	68.643 (HiFi)	34184
36_pra_6	148673945	435	27563271	55.3956 (HiFi)	41168
37_pu_2_23	153157137	409	33197217	54.5193 (HiFi)	40921
38_dra_2	147763393	220	27052895	57.7587 (HiFi)	38924
39_ah_7	142198232	165	26281650	60.2447 (HiFi)	36737
40_etna_2	160472342	488	27571386	57.7138 (HiFi)	40522
41_sorbo	156140576	682	25801151	55.3755 (HiFi)	44294
42_arb_0	152406383	509	27077471	56.8016 (HiFi)	41851
43_elk_1	156188303	631	27089323	55.5216 (HiFi)	42445
44_ket_10	162501778	559	27867807	55.2915 (HiFi)	43372
45_meh_0	152472945	576	24217380	56.226 (HiFi)	40821
ARAPORT11	119668634	7	23459830	45.8899 (HiFi)	33243

The publicly available HiFi data for all accessions were downloaded for assembly, as well as the Illumina data for QA of two accessions (CNCB BioProject PRJCA012695) and Hi-C data for one accession (SRR20242239), which was used for guiding hifiasm as well as YaHS. All accessions were assembled into 51 to 819 contigs and the pipeline was able to scaffold and rename all but one accession relative to the five chromosomes of the *A. thaliana* TAIR10 assembly (Supplementary Table 1). The resulting assemblies had a QV between 50 - 70 given their input HiFi data, indicating high accuracy. Annotation by LiftOff and Helixer resulted in 32,711 - 47,793 predicted gene models with a BUSCO completeness between 91.9% and 99.9%.

Although the resulting assemblies were complete and largely congruent with expectations, they were not identical to the findings of Kang et al. (2023). Three accessions (Yilong, SB30 and Tibet) had notably larger numbers of contigs and predicted genes, which seemed to correlate with the amount of potential contamination in these assemblies (as revealed by the Kraken2 and FCS-GX analyses) (Supplementary Figure 1). Only unscaffolded contigs were labelled as contaminated, and the total number of genes on only the five chromosomes per accession was 29,663 - 34,621. This number closely matched the expected number of genes in ARAPORT11 (33,243), indicating that the scaffolds themselves were likely free of contamination. Therefore, it is likely that Kang et al. (2023) performed contamination filtering prior to assembly, though undocumented.

The assemblies from MoGAAAP could not immediately be used for a pangenome study, because they still require human correction. For example, analysing the Hi-C contact maps of the Col-0 assembly indicated a mistake in scaffolding the chromosome 1 by ntJoin (Supplementary Figure 2), which occurred after Hi-C-based scaffolding by YaHS. Comparison of the MUMmerplot and Hi-C plot for Col-0 confirmed the incorrect assembly for this contig. Such inconsistencies need to be manually resolved by cutting the assembly and manually joining the correct sequences together. In summary, the automated QA statistics and figures were very helpful in finding and solving mistakes in an informed manner.

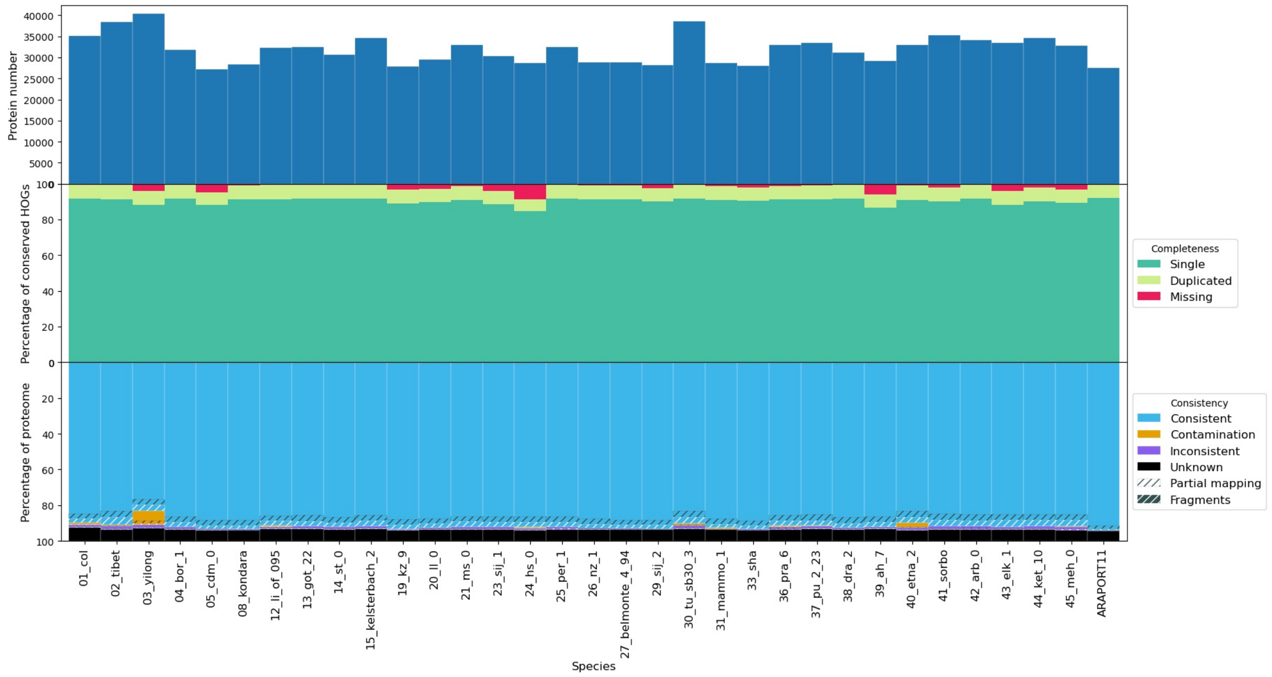

Supplementary Figure 1: OMArk result of analysing the proteomes of the provisional annotations for the 32 *A. thaliana* created by MoGAAAP. Most proteomes are very complete and consistent, and have about the same number of ∼30,000 proteins. However, some proteomes that were larger showed clear indications of contamination (therefore, genes predicted in non-*A. thaliana* sequences).

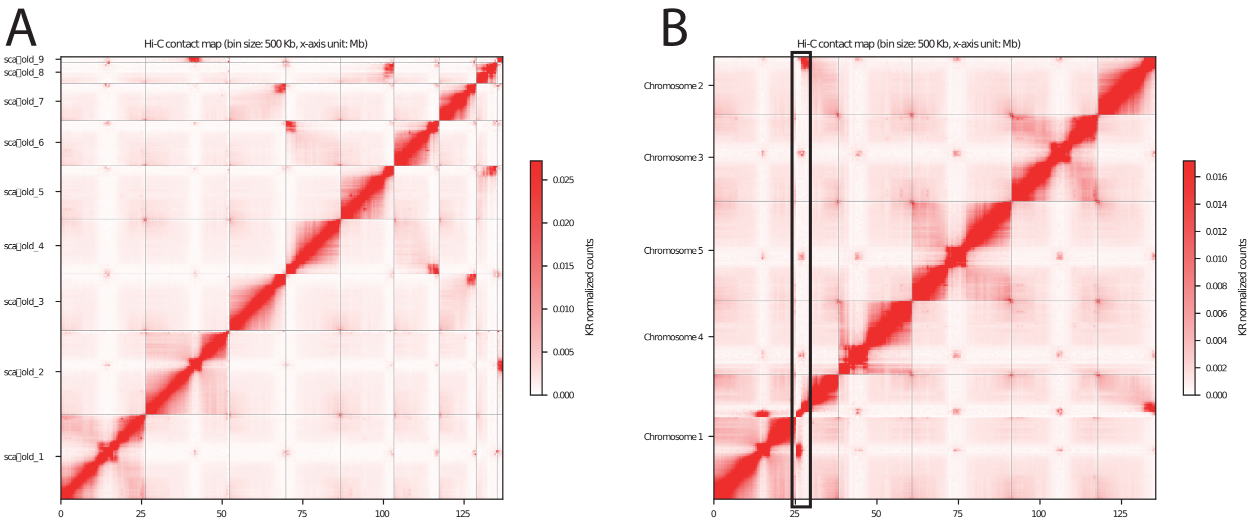

Supplementary Figure 2: Evidence of a scaffolding mistake in the *A. thaliana* Col-0 assembly for chromosome 1. The mistake in second half part of chromosome 1 is highlighted in the figure by a black rectangle. A) The scaffolding of the hifiasm contigs based on Hi-C data by YaHS. B) The reference-guided scaffolding by ntJoin based on the TAIR10 assembly with this scaffolding mistake indicated by a black rectangle.

Grapevine

Grapevine is a highly heterozygous diploid plant species that was recently used for a pangenome study in which novel accessions were sequenced with HiFi, ONT and Hi-C (Liu et al., 2024). We assembled six publicly available accessions using MoGAAAP employing all three data types, and succeeded in obtaining separate haplotypes from the hifiasm assembly.

Supplementary Table 2: Key statistics of the genomes included in the grapevine pangenome from MoGAAAP.

Name	Total length	#sequences	N50	Total QV	#genes
Baimunage.hap1	504881989	276	25118660	52.4828 (HiFi)	32720
Baimunage.hap2	509837772	205	25618253	52.5164 (HiFi)	32324
Hongmunage.hap1	597416486	597	24578386	57.8767 (HiFi)	49375
Hongmunage.hap2	512639455	317	25019525	61.6364 (HiFi)	36099
Manicure_Finger.hap1	496712247	90	25106930	57.7527 (HiFi)	32978
Manicure_Finger.hap2	492760379	64	25283662	64.5285 (HiFi)	31932
Muscat_Hamburg.hap1	515037590	262	26148634	58.13 (HiFi)	38977
Muscat_Hamburg.hap2	501470328	127	24870047	61.4175 (HiFi)	34332
Shine_Muscat.hap1	508312460	131	25342229	63.0682 (HiFi)	31728
Shine_Muscat.hap2	489297438	106	24667827	60.9506 (HiFi)	31515
Wolley.hap1	556836588	285	28216695	60.5776 (HiFi)	38900
Wolley.hap2	542898371	115	27633440	64.0357 (HiFi)	33346

All chromosome statistics closely matched the values reported by Liu et al. (2024) without any manual correction (Supplementary Table 2). Two haplotypes were assembled for each chromosome by the hifiasm assembler and all had a QV of 50 to 65, indicating high accuracy. However, the comparison also revealed some differences. The MUMmerplots identified a potential scaffolding error where two chromosomes had been joined

in one of the two Hongmunage haplotypes, which was confirmed in the Hi-C contact map. This difference is likely caused by a different scaffolding algorithm: Liu et al. (2024) used RagTag whereas MoGAAAP uses ntJoin by default. Also, the BUSCO score for one of the Shine Muscat haplotypes from MoGAAAP was found to be 0. Looking into this problem, it turned out to be caused by the BUSCO software as Helixer had annotated a protein larger than 100,000 amino acids, which is not allowed by BUSCO.

Upon closer inspection of the QA report from MoGAAAP, some contamination problems of the grapevine reads became obvious that were previously not reported by Liu et al. (2024). Hongmunage was also the only accession with a large amount of contamination as identified by FCS-GX, Kraken2 and OMArk. This was also reflected by the large number of genes on haplotype 1 (49,375) of this accession versus all other grapevine haplotypes (∼31,000 to ∼39,000) as well as the large amount of unscaffolded sequence. According to FCS-GX and Kraken2, the main contaminants were *Erisyphe necator* (grape powdery mildew) and insect. Interestingly, the contamination was not mentioned by Liu et al. (2024) even though a clean assembly was reported without a significantly larger number of genes or unscaffolded contigs. The amount of contamination by grape powdery mildew was so large that it showed up in the k-mer spectra plots as well, which indicated it was assembled in almost its entirety as part of haplotype 1 of Hongmunage (Supplementary Figure 3). One way to resolve this issue would therefore be to clean the reads of Hongmunage of *Erisyphe necator* and insect reads and assemble it again, but to keep the assemblies and annotations as generated by MoGAAAP for all other accessions. Cleaning the reads of Hongmunage could for example be done by aligning them to the contaminant assemblies or running kraken2 on the reads and filtering out all contaminant reads.

Human trio

MoGAAAP is not only designed for plants but for any eukaryotic genome. In order to demonstrate applicability to non-plant species, we applied the pipeline to a human dataset. With the rise of pangenomics, it is important to have a pipeline that can ensure consistency in assembly and structural annotation. We therefore assembled the HiFi data of one of the trios of the human pangenome project: HG002, HG003 and HG004. In addition, for HG002, we also used Hi-C data to show its integration in the pipeline.

Supplementary Table 3: Key statistics of the genomes included in the human trio use case with MoGAAAP.

Name	Total length	#sequences	N50	Total QV	#genes
HG002.hap1	3136141629	374	144486466	67.256 (HiFi)	86255
HG002.hap2	2862415627	216	145663935	67.1341 (HiFi)	80961
HG003.hap1	3009685137	260	145333704	66.444 (HiFi)	84045
HG003.hap2	3030172493	233	146486266	66.3156 (HiFi)	83764
HG004.hap1	3047232247	192	152205959	66.1976 (HiFi)	84514
HG004.hap2	3015960278	188	151882972	65.9926 (HiFi)	84704

After hifiasm assembly, reference-guided scaffolding with ntJoin was able to put 95.5% - 97.5% of the assembled length into chromosomes based on the GRCh38 reference genome for each haplotype (Supplementary Table 3. QA was performed on all haplotypes separately. Confirming the accuracy of the scaffolding, the MUMmerplot figures showed full collinearity for all chromosomes; except for the mother HG004 which lacked a Y chromosome as expected (Supplementary Figure 4), and X chromosomes of the son and father which contain gaps compared to the GRCh38 reference genome. Furthermore, the contact map generated with the Hi-C data showed no miss-scaffolding for HG002 (Supplementary Figure 5).

The QV as calculated by merqury based on the HiFi data showed high k-mer completeness (66.0 - 67.3) for each haplotype. Correspondingly, BUSCO and OMArk showed full completeness of the assembly and

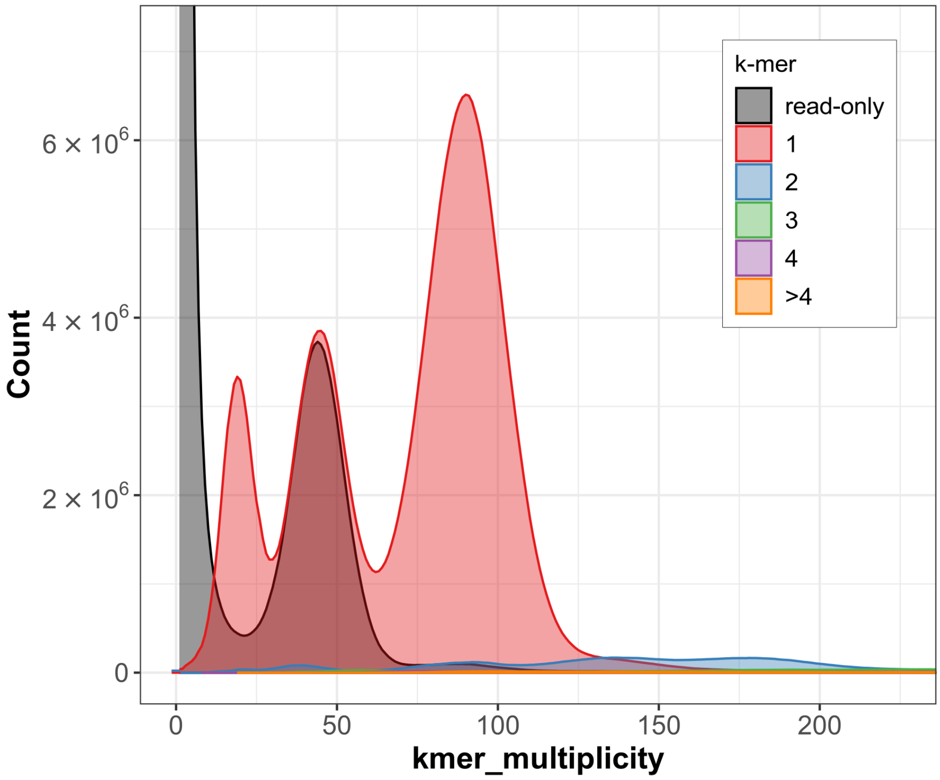

Supplementary Figure 3: K-mer spectra-CN plot created by merqury for the HiFi reads of grapevine cultivar Hongmunage against the haplotype 1 assembly of this accession. Strangely, three red peaks (showing HiFi k-mers that occur once in the assembly) are visible instead of the expected two. The right-most peak represents the k-mers present in both haplotypes of Hongmunage, the middle peak represents the k-mers that are haplotype-specific in Hongmunage (at half multiplicity compared to the right-most peak), and the left-most peak represents the k-mers of a large contaminant species present in the HiFi data (likely grape powdery mildew).

annotation (95% - 98% and 94% - 97%, respectively). Confirming previous work on these cell lines, only human gammaherpesvirus 4 (also known as Epstein-Barr virus (EBV)) contamination was found in the assemblies using both Kraken2 and FCS-GX. EBV was used to immortalise the cells and therefore expected to still be present genomically (Zook et al., 2016). This analysis shows that non-plant species may also be assembled, scaffolded, analysed, annotated and assessed for quality using MoGAAAP.

Silkmoth

Demonstrating applicability to invertebrate animals, we successfully applied MoGAAAP for the assembly, annotation and QA of a silkmoth genome. Based on a combination of HiFi and Hi-C data from Wan et al. (2025), MoGAAAP constructed a chromosome-level assembly in which 20,397 genes were annotated (of which 15,389 coding), slightly higher than the reference genome in NCBI (Supplementary Table 4). Merqury computed a QV score of 68.8, neither kraken2 nor FCS-GX identified any contamination and BUSCO and OMArk indicated high gene completeness of both assembly and preliminary annotation.

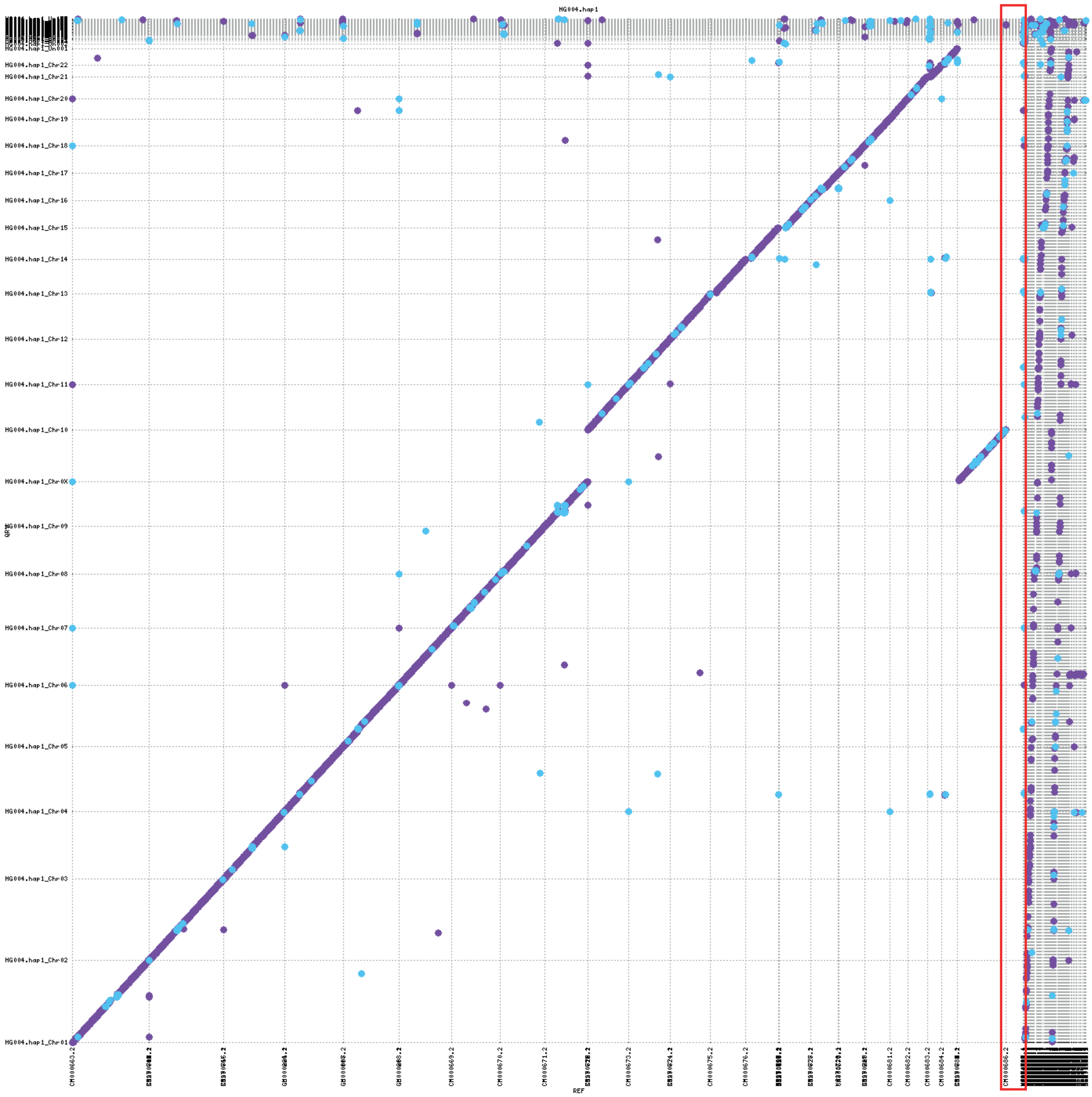

Supplementary Figure 4: MUMmerplot of the first haplotype of the HG004 (mother) against the GRCh38 reference genome. The lack of alignment at the Y chromosome (highlighted in red) indicates this individual only has an X chromosome.

Supplementary Table 4: Key statistics of silkworm use case with MoGAAAP.

Name	Total length	#sequences	N50	Total QV	#genes
KA	459494510	50	16704091	68.8834 (HiFi)	20397

Yeast

Finally, we assessed applicability of MoGAAAP to the fungal kingdom using yeast as an example. Reusing the HiFi data from the strain CICC-1445 from Zhang et al. (2022), we obtained two haplotypes of 16 chromosomes

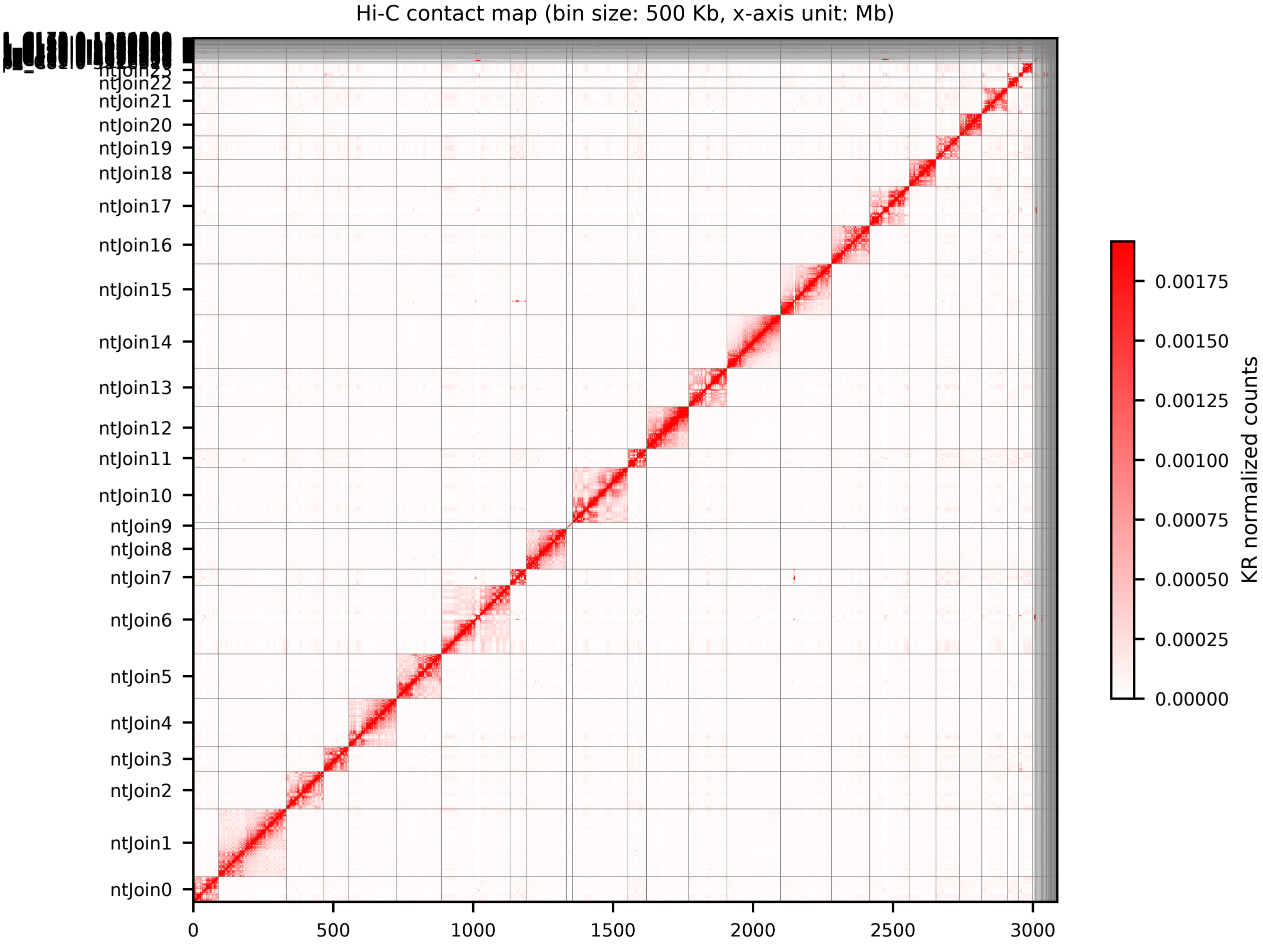

Supplementary Figure 5: Hi-C contact map of one of the two scaffolded HG002 haplotypes, which confirms the accuracy of scaffolding.

(Supplementary Table 5). While Merqury found a relatively high completeness (48.8 and 49.7 for each haplotype overall and infinity (perfect) for many chromosomes), inspection of the alignment to the R64 reference genome indicated potential misassemblies by hifiasm: some chromosomes had large duplicated sections that were joined by scaffolding (Supplementary Figure 6). Since the duplication problem is already present in the contigs, potentially hifiasm was not able to accurately separate the two haplotypes. Without the availability of Hi-C data for this strain to aid hifiasm assembly, potentially 'purge_dups' (Guan et al., 2020) could resolve the issue by purging the duplicated contigs. Despite the contigging problems, MoGAAAP finished successfully and produced the report that helped in uncovering these assembly difficulties.

Supplementary Table 5: Key statistics of yeast use case with MoGAAAP.

Name	Total length	#sequences	N50	Total QV	#genes
CICC1445.hap1	17244564	51	925691	48.8137 (HiFi)	8193
CICC1445.hap2	17592386	30	1038062	49.7629 (HiFi)	9033

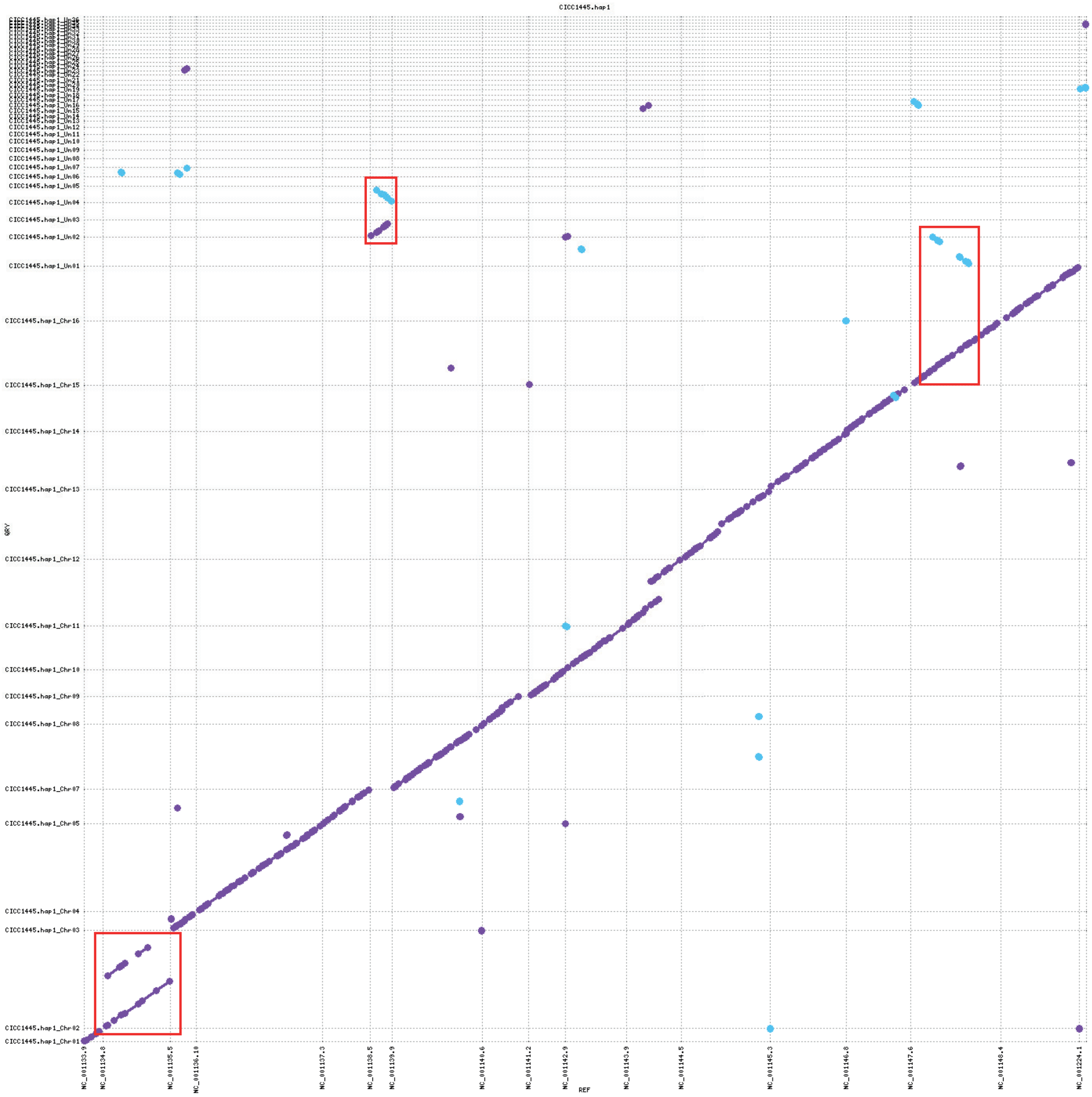

Supplementary Figure 6: MUMmerplot of the first assembled haplotype of *Saccharomyces cerevisiae* CICC-1445 aligned against the R64 reference genome. Duplicated sequences in the CICC-1445 assembly can be seen for some chromosomes compared to R64 (highlighted with red boxes).

Application

To show that MoGAAAP, in combination with manual curation, can be used for the creation and assessment of a publication-quality genome, we applied it to a novel *Lactuca serriola* genome.

Lactuca serriola

L. serriola is the likely progenitor of cultivated lettuce (*Lactuca sativa*) and is of major importance for lettuce breeding as a source of wild alleles (Wei et al., 2021). No high-quality genome assembly has been published for *L. serriola*. Therefore, we used our pipeline to assemble the genome of *L. serriola* (US96UC23) using HiFi and ONT reads and scaffold against a high-quality *L. sativa* genome as the two species are closely related.

Sequencing information

L. serriola (US96UC23) seedlings were grown in Magenta boxes under sterile conditions in the dark. Seven day old etiolated seedlings were harvested and flash frozen in liquid nitrogen. Etiolated tissue was used for DNA extraction using a CTAB DNA extraction/sorbitol cleanup protocol. The HiFi library was prepared using PacBio SMRTbell prep kit 3.0 followed by bead clean up and size selection (>13.6 kb). HiFi sequencing was performed using the PacBio Revio platform aiming for 20x coverage. In total 56.9 Gb of HiFi reads were generated. ONT libraries were prepared with the LSK109 kit. ONT sequencing was performed using PromethION on an R9.4.1 chip aiming for 140x coverage and base called with Guppy (v5.0.16.sup). 10.8M reads (total 248.8 Gb) were generated, after which Porechop v0.2.3 was used to remove residual ONT adapters and NanoFilt v2.7.1 was used to select reads with an average quality score >Q10.

Genome assembly

Genome assembly was performed using hifiasm with all HiFi reads and a subset of the ONT reads, specifically only reads > 36kb. In total, we used 56.9 Gb of HiFi reads and 84.0 Gb of ultra-long ONT reads, corresponding to an expected coverage of 21.9x and 32.3x coverage respectively. The resulting contig assembly size of 2.559 Gb was consistent with the expected genome size of 2.6 Gb. In order to scaffold the 258 contigs relative to the *L. sativa* Salinas v11 RefSeq genome (GCF_002870075.4) (Reyes-Chin-Wo et al., 2017), we had to relax the default ntJoin parameters for window length to 40,000 bp (with $k = 52$), because *L. serriola* is a different species than *L. sativa*. Failing to do so caused chromosome joining. Scaffolding resulted in 29 contigs scaffolded into nine pseudomolecules, making up 99.35% of total assembly length. The remaining 229 contigs (0.65% of total assembly length) could not be scaffolded. Of these, 92 were identified as organellar contigs.

Annotation

Next, the pipeline performed the lifting over of genes from v11 of *L. sativa* Salinas RefSeq genome annotation. LiftOff was able to transfer the coordinates of 40,254 genes (of which 29,693 were protein-coding). This was supplemented with 9,283 non-overlapping gene models from Helixer. In total, this resulted in 38,976 protein-coding genes. This is in the same range as other annotated *Lactuca* sp.: *L. sativa* Salinas has 36,855, *Lactuca saligna* CGN05327 has 42,908 and *Lactuca virosa* CGN04683 has 39,887 protein-coding genes annotated.

Quality assessment

Based on an independent Illumina short-read sequencing dataset (CNS0047707) of the same accession by Wei et al. (2021), the QV value of the assembly was 50.5 (separate chromosomes ranging from [52 - 56]) and the assembly was 99.06% complete. Also, the spectra-CN plot with the independent Illumina short-read data confirmed a correct assembly: no read- or assembly-only k-mers were identified. BUSCO and OMArk scores confirmed the high completeness of the assembly and annotation with values of 98.2% and 97.8% respectively. The extensive contamination screening by the pipeline (OMArk, Kraken2, FCS-GX) was not able to find any non-lettuce sequence, highlighting the robust library preparation. Interestingly, a large inversion on the right

arm of chromosome 1 with respect to *L. sativa* Salinas v11 was identified in the MUMmerplot. The location of this ∼24Mb inversion was consistent with the genetic map previously published by Reyes-Chin-Wo et al. (2017) (see their Supplementary Figure 9) which is a cross between *L. sativa* Salinas and the here assembled *L. serriola* US96UC23. For validation, we used the alignment of both HiFi and ONT long-read data to confirm the correctness of the assembly (Supplementary Figure 7).

GenBank submission

The scaffolded assembly and annotation were prepared for submission to GenBank, which involved file formatting and a final confirming that they were of high quality (free of contamination, complete and accurate). During this iterative process, both assembly and annotation had to be slightly adjusted. We used GAG (Geib et al., 2018) to convert the files to tbl format and identify potential issues. For the assembly, we took the nuclear assembly and shortened the sequence headers. For the annotation, we first renamed all genes to remove traces of the gene identifiers from *L. sativa* Salinas v11, retained only the longest isoform per gene and removed all non-coding genes. Some genes had overlapping CDS sequences on opposing strands; for these, the shortest of each pair was removed. Finally, we removed all genes without start or stop codons and all genes with introns smaller than 10 nucleotides because these are not permitted by GenBank. After the cleaning process, 37,518 protein-coding genes remained.

The *L. serriola* assembly can be found at GenBank GCA_051521515.1. The underlying HiFi and ONT reads have been released under BioProject PRJNA412928.

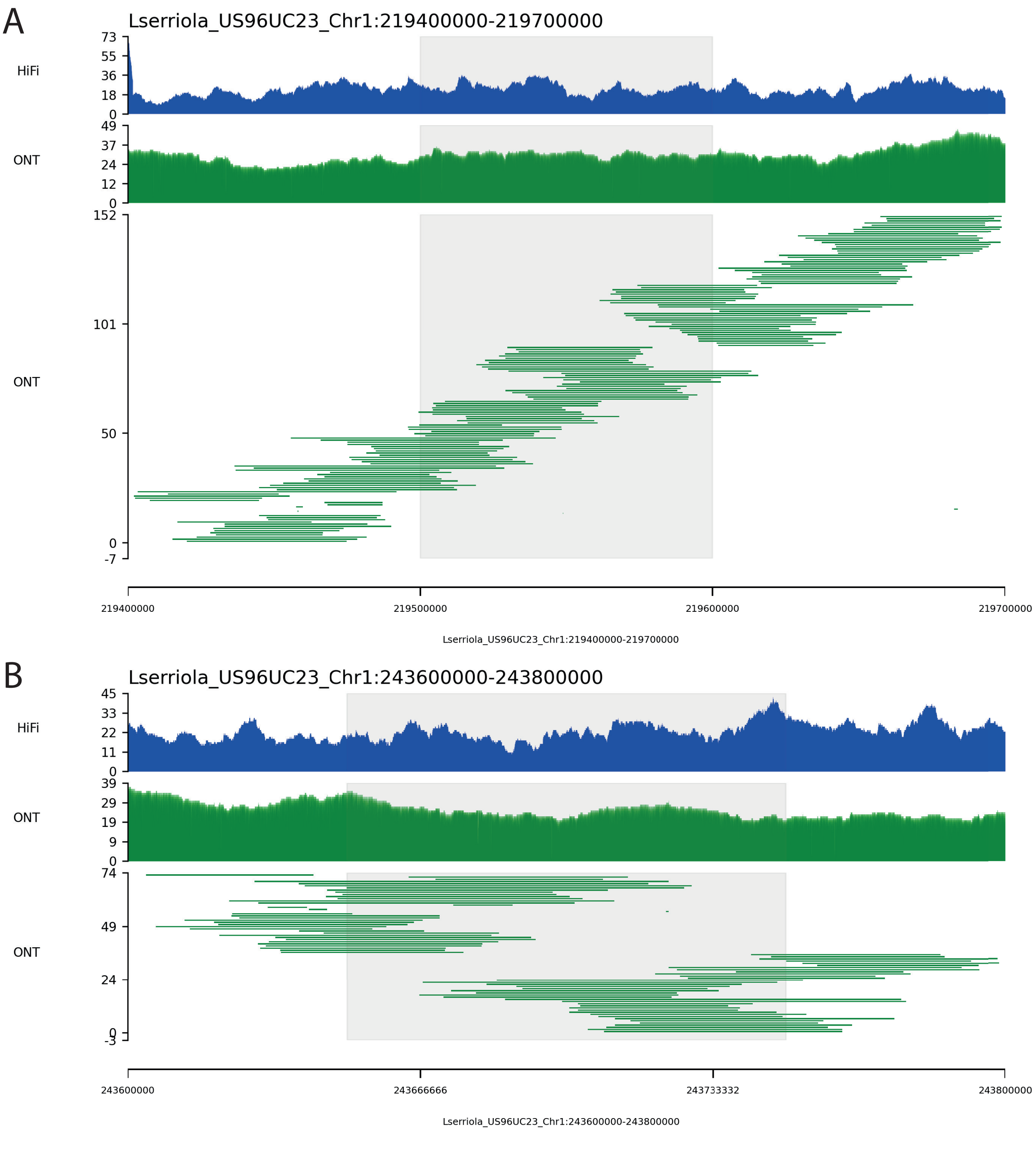

Supplementary Figure 7: The left (A) and right (B) side of the ∼24Mb inversion of *L. serriola* UC96UC23 compared to *L. sativa* Salinas v11 (the location of the breakpoint is highlighted in gray). Coverage is plotted for both HiFi and ONT (top two tracks) and all individual ONT reads fully within the region are shown in the third track. Since neither HiFi nor ONT shows any drop in coverage or breakpoint, the *L. serriola* assembly is structurally correct and the inversion real.

References

Geib, S. M., Hall, B., Derego, T., Bremer, F. T., Cannoles, K., & Sim, S. B. (2018). Genome Annotation Generator: A simple tool for generating and correcting WGS annotation tables for NCBI submission. *GigaScience*, *7*(4), giy018. https://doi.org/10.1093/gigascience/giy018

Guan, D., McCarthy, S. A., Wood, J., Howe, K., Wang, Y., & Durbin, R. (2020). Identifying and removing haplotypic duplication in primary genome assemblies. *Bioinformatics*, *36*(9), 2896–2898. https://doi.org/10.1093/bioinformatics/btaa025

Kang, M., Wu, H., Liu, H., Liu, W., Zhu, M., Han, Y., Liu, W., Chen, C., Song, Y., Tan, L., Yin, K., Zhao, Y., Yan, Z., Lou, S., Zan, Y., & Liu, J. (2023). The pan-genome and local adaptation of *Arabidopsis thaliana*. *Nature Communications*, *14*(1), 6259. https://doi.org/10.1038/s41467-023-42029-4

Liu, Z., Wang, N., Su, Y., Long, Q., Peng, Y., Shangguan, L., Zhang, F., Cao, S., Wang, X., Ge, M., Xue, H., Ma, Z., Liu, W., Xu, X., Li, C., Cao, X., Ahmad, B., Su, X., Liu, Y., … Zhou, Y. (2024). Grapevine pangenome facilitates trait genetics and genomic breeding. *Nature Genetics*, 1–11. https://doi.org/10.1038/s41588-024-01967-5

Reyes-Chin-Wo, S., Wang, Z., Yang, X., Kozik, A., Arikit, S., Song, C., Xia, L., Froenicke, L., Lavelle, D. O., Truco, M.-J., Xia, R., Zhu, S., Xu, C., Xu, H., Xu, X., Cox, K., Korf, I., Meyers, B. C., & Michelmore, R. W. (2017). Genome assembly with in vitro proximity ligation data and whole-genome triplication in lettuce. *Nature Communications*, *8*(1), 14953. https://doi.org/10.1038/ncomms14953

Wan, L., Deng, C., Liu, B., Su, S., Zhang, Z., Jiang, Y., Zou, B., Liu, J., Du, Z., Zhang, Y., Chen, P., & Xiao, W. (2025). Telomere-to-telomere genome assemblies of three silkworm strains with long-term pupal characteristics [Publisher: Nature Publishing Group]. *Scientific Data*, *12*(1), 501. https://doi.org/10.1038/s41597-025-04860-w

Wei, T., van Treuren, R., Liu, X., Zhang, Z., Chen, J., Liu, Y., Dong, S., Sun, P., Yang, T., Lan, T., Wang, X., Xiong, Z., Liu, Y., Wei, J., Lu, H., Han, S., Chen, J. C., Ni, X., Wang, J., … Liu, H. (2021). Whole-genome resequencing of 445 *Lactuca* accessions reveals the domestication history of cultivated lettuce. *Nature Genetics*, *53*(5), 752–760. https://doi.org/10.1038/s41588-021-00831-0

Zhang, X., Liu, C.-G., Yang, S.-H., Wang, X., Bai, F.-W., & Wang, Z. (2022). Benchmarking of long-read sequencing, assemblers and polishers for yeast genome. *Briefings in Bioinformatics*, *23*(3), bbac146. https://doi.org/10.1093/bib/bbac146

Zook, J. M., Catoe, D., McDaniel, J., Vang, L., Spies, N., Sidow, A., Weng, Z., Liu, Y., Mason, C. E., Alexander, N., Henaff, E., McIntyre, A. B. R., Chandramohan, D., Chen, F., Jaeger, E., Moshrefi, A., Pham, K., Stedman, W., Liang, T., … Salit, M. (2016). Extensive sequencing of seven human genomes to characterize benchmark reference materials. *Scientific Data*, *3*(1), 160025. https://doi.org/10.1038/sdata.2016.25
